# Cardio-Thoracic Ratio Is Stable, Reproducible and Has Potential as a Screening Tool for HIV-1 Related Cardiac Disorders in Resource Poor Settings

**DOI:** 10.1371/journal.pone.0163490

**Published:** 2016-10-04

**Authors:** Hanif Esmail, Tolu Oni, Friedrich Thienemann, Nashreen Omar-Davies, Robert J. Wilkinson, Mpiko Ntsekhe

**Affiliations:** 1 Institute of Infectious Diseases and Molecular Medicine, Clinical Infectious Disease Research Initiative, University of Cape Town, Cape Town, South Africa; 2 Department of Medicine, Imperial College London, London, United Kingdom; 3 Radcliffe Department of Medicine, University of Oxford, Oxford, United Kingdom; 4 School of Public Health and Family Medicine, Division of Public Health Medicine, University of Cape Town, Cape Town, South Africa; 5 Department of Medicine, University of Cape Town, Cape Town, South Africa; 6 The Francis Crick Institute, Mill Hill Laboratory, London, United Kingdom; 7 Department of Medicine, Division of Cardiology, University of Cape Town, Cape Town, South Africa; Azienda Ospedaliero Universitaria Careggi, ITALY

## Abstract

**Background:**

Cardiovascular disorders are common in HIV-1 infected persons in Africa and presentation is often insidious. Development of screening algorithms for cardiovascular disorders appropriate to a resource-constrained setting could facilitate timely referral. Cardiothoracic ratio (CTR) on chest radiograph (CXR) has been suggested as a potential screening tool but little is known about its reproducibility and stability. Our primary aim was to evaluate the stability and the inter-observer variability of CTR in HIV-1 infected outpatients. We further evaluated the prevalence of cardiomegaly (CTR≥0.5) and its relationship with other risk factors in this population.

**Methodology:**

HIV-1 infected participants were identified during screening for a tuberculosis vaccine trial in Khayelitsha, South Africa between August 2011 and April 2012. Participants had a digital posterior-anterior CXR performed as well as history, examination and baseline observations. CXRs were viewed using OsiriX software and CTR calculated using digital callipers.

**Results:**

450 HIV-1-infected adults were evaluated, median age 34 years (IQR 30–40) with a CD4 count 566/mm^3^ (IQR 443–724), 70% on antiretroviral therapy (ART). The prevalence of cardiomegaly was 12.7% (95% C.I. 9.6%-15.8%). CTR was calculated by a 2^nd^ reader for 113 participants, measurements were highly correlated r = 0.95 (95% C.I. 0.93–0.97) and agreement of cardiomegaly substantial **κ** = 0.78 (95% C.I 0.61–0.95). CXR were repeated in 51 participants at 4–12 weeks, CTR measurements between the 2 time points were highly correlated r = 0.77 (95% C.I 0.68–0.88) and agreement of cardiomegaly excellent **κ** = 0.92 (95% C.I. 0.77–1). Participants with cardiomegaly had a higher median BMI (31.3; IQR 27.4–37.4) versus 26.9; IQR 23.2–32.4); p<0.0001) and median systolic blood pressure (130; IQR 121–141 versus 125; IQR 117–135; p = 0.01).

**Conclusion:**

CTR is a robust measurement, stable over time with substantial inter-observer agreement. A prospective study evaluating utility of CXR to identify cardiovascular disorder in this population is warranted.

## Introduction

The rising impact on health of non-communicable diseases (NCD) (mainly cardiovascular disease, cancer, diabetes and chronic respiratory disease) in low and middle income countries (LMIC) is of great concern and implementation of measures to curb this is high on the global health agenda. Co-ordinated strategies that strengthen health systems and the capacity of primary care to prevent and detect these chronic diseases early are a key component of the response[1]. In sub-Saharan Africa (SSA) the HIV-1 epidemic directly impacts on NCD burden. HIV-1 predisposes to cardiovascular disorders through the direct effects of HIV-1, HIV-1 related immune-activation and inflammation, opportunistic infections, drug toxicity and metabolic consequences of antiretroviral therapy (ART) in addition to other cardiovascular risk factors that disproportionately affect this population[2, 3].

In a recent study characterising presentations of 518 HIV-1 infected adults to a tertiary cardiac unit in South Africa, cardiomyopathy was the commonest diagnosis accounting for 38% of presentations (structural dilatation was present in over 50%) followed by pericarditis/pericardial effusion in 24%. 8% of patients had evidence of pulmonary hypertension and of note, the majority of patients were on ART at presentation[4]. In SSA and similar resource-limited settings, access to such specialist cardiology services is constrained and misdiagnosis in primary care may contribute to delayed referral and advanced presentation explaining in part, the poor prognosis associated with HIV-associated cardiac disease. Although early use of ART is believed to have resulted in a reduced incidence of these cardiac complications to an extent [5], screening for cardiac disease is crucial to allow for early detection and appropriate specific therapy in order to prevent progression and improve survival.

Simple, cost effective screening tools and strategies that assist timely referral of these patients for appropriate assessment are needed. Cardiothoracic ratio (CTR) on chest radiograph (CXR), the ratio of maximal cardiac diameter to internal thoracic diameter, with CTR≥0.5 usually considered abnormal, has previously been proposed as a potential screening tool in this setting[6, 7]. In a tertiary referral hospital in Botswana, 106 HIV-1 infected patients (median CD4 299, 54% on ART) with cardiomegaly on CXR (all of whom had CTR≥0.53) underwent echocardiography, 99% had an abnormal echocardiogram (41% cardiomyopathy, 29% pericarditis, 9% right heart failure) [6]. A second study set in a tertiary referral hospital in South Africa evaluated 41 HIV-1 infected individuals with TB pericarditis and pericardial effusions > 10mm and demonstrated that all had CTR≥0.55[7]. However, such studies are prone to referral bias and therefore cannot be used to determine sensitivity or specificity of particular CTR cut offs, this would necessitate evaluation in a variety of asymptomatic persons in primary care settings.

Chest radiography has been a widely available investigation for decades and cardiomegaly has been shown repeatedly to be predictive of cardiovascular mortality in several large studies[8, 9]. However CTR has been shown to have a limited role as a screening tool for left ventricular failure as approximately a third of patients with Left Ventricular Ejection Fraction (LVEF) <0.35 will have a CTR < 0.5[10]. This is in part as the left ventricle is not the primary contributor to the cardiac shadow and not all those with left ventricular failure will have enlarged ventricles, as a result, correlation of CTR with LVEF can be weak[10–12]. Correlation of transverse diameter of the heart shadow on CXR with Left Ventricular End Diastolic Volume (LVEDV) has been found to be good [13] and in addition CTR correlates better with right ventricular and atrial size than for left ventricular size[14]. CTR may therefore be more useful in HIV-1 infected population especially in SSA as a screening tool for the common cardiac presentations in this setting rather than a narrow screen for left ventricular dysfunction, as dilated cardiomyopathy and pulmonary artery hypertension and pericardial effusions are more likely to cause elevated CTR.

Before CTR can be evaluated for its predictive value as a screening tool for cardiovascular abnormalities in HIV-1, as for any test, it is important to first establish if CTR is a stable and reproducible measure. In particular for an imaging test of this nature, variability can be created through biological factors such as cardiac motion and depth of inspiration, technical factors such as patient positioning or machine characteristics and inter-observer variability[15]. All these factors could affect the reproducibility of CTR making it an unsuitable screening tool.

As a preliminary evaluation of CTR to inform future studies in this area, the primary aim of our study was to evaluate the inter-observer variability and the reproducibility of CTR in this population over time.

## Materials and Methods

The study adhered to International Conference on Harmonisation Good Clinical Practice guidelines, and was approved by the University of Cape Town's Faculty of Health Sciences Human Research Ethics Committee (REC 001/2010). All participants provided written informed consent.

Participants were identified between August 2011 and April 2012 during screening for a phase IIB tuberculosis vaccine trial[16]. All participants were resident in Khayelitsha, a peri-urban township of Cape Town, South Africa. Screening investigations included a digital posterior-anterior CXR (PA-CXR), 2 sputum cultures for *Mycobacterium tuberculosis* (Mtb), as well as history, examination, baseline observations and basic demographic details. As per-protocol, a proportion of the participants had repeat CXR performed after approximately 6–12 weeks to ensure no progressive parenchymal lesions thought to be due to either active or inactive tuberculosis. All study data were collected on electronic case report forms (web-based platform) and stored on a dedicated secure central database.

Inclusion criteria for the study were healthy HIV-1-infected patients, aged 18–50 years either established on ART or ART-naïve with CD4 ≥ 300/mm^3^. Participants were excluded if they had culture positive sputum for *Mycobacterium tuberculosis*, if less than 8 posterior ribs were visible on CXR or if mediastinal shift was present from fibrosis relating to previous TB.

PA-CXR in full inspiration with arms pronated were performed at the beginning of the study on a Delft Odelca DR and then subsequently on a Phillips Essenta DR at 125kV with source to image distance 180cm. Digital images were uploaded using OsiriX software and viewed on a 2 Megapixel screen in low ambient light.

**Fig 1:**
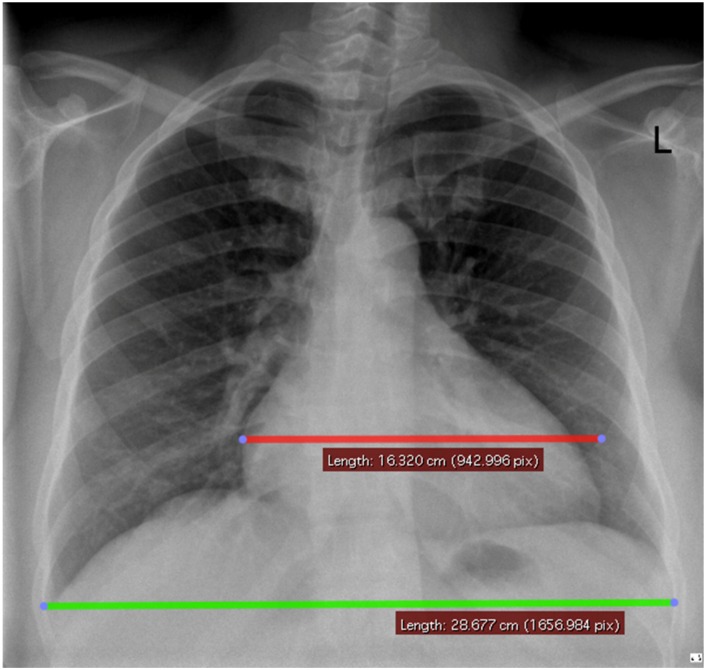
Method for determining Cardiothoracic Ratio. Digital Posterior-Anterior Chest Radiograph (CXR) with maximal cardiac diameter in red and maximal thoracic diameter in green. Cardiothoracic ratio (CTR) is 0.57.

CTR was calculated as ratio of maximal width of cardiac shadow to maximal internal thoracic width visible on CXR using digital callipers (Fig 1). A CTR ≥ 0.50 was considered cardiomegaly. A second reader independently evaluated 113 of 450 (25%) baseline CXR (systematically identified as every 4^th^ CXR chronologically) without knowledge of primary readers results. Sample size was determined using methodology described by Cantor *et al* [17]. Assuming 50% inter-reader agreement and a 20% relative error margin a sample size of at least 100 was required to achieve power of 80% at α = 0.05. 51 participants had repeat CXR as per-protocol, CTR for the repeat CXR was evaluated by the primary reader without reference to the initial CXR.

### Statistical analysis plan

The Shapiro-Wilk test was used to establish normality of data. Correlation of CTR measurements was then calculated by Pearson’s or Spearman’s coefficients as appropriate. Agreement between observers as to which participants had cardiomegaly (CTR ≥ 0.50) was determined using the Kappa test statistic and 95% confidence intervals calculated. Non-paired, non-parametric data were compared using Wilcoxon rank sum (Mann-Whitney *U*) test and parametric data using student’s t-test. Proportions were compared by χ^2^ test. Risk factors associated with cardiomyopathy were analysed using logistic regression. The model was built manually using a bidirectional stepwise regression approach, sequentially including variables with a p value <0.20 in univariate analyses. All data was analysed using STATA 12.0 (StataCorp, College Station, TX, USA).

## Results

483 participants had CXR available, 20 were excluded, as culture positive for tuberculosis, 5 excluded with suboptimal CXR and 8 had no clinical information available. 450 HIV-1-infected adults were included with median age 34 years (IQR 30–40), 86% female, median CD4 count 566 (IQR 443–724) with 70% prescribed ART. All participants were Black African, specific ethnicity was not inquired about, but at last census 97% of Khayelitsha residents were Xhosa. The prevalence of cardiomegaly in this group was 12.7% (95% C.I. 9.6%-15.8%) with 3.1% having CTR ≥ 0.53 and 1.8% having CTR ≥ 0.55.

### Inter-reader correlation

**Fig 2:**
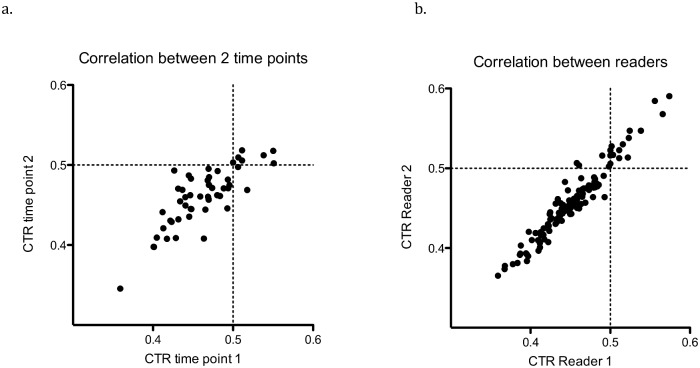
Correlation between timepoints and readers. a. Scatter plot showing correlation of cardiothoracic Ratio (CTR) for 51 participants with repeat chest radiograph (CXR) 4–12 weeks apart read by a single reader. Top left and bottom right quadrants represent differences in classification of cardiomegaly between reads. b. Scatter plot showing correlation of CTR for 113 participants determined independently by 2 readers. Top left and bottom right quadrants represent differences in classification of cardiomegaly between readers.

CTR was calculated by a 2^nd^ reader for 113 of 450 participants. The independent measurements of CTR by the 2 readers were highly correlated r = 0.95 (95% C.I. 0.93–0.97) p<0.0001. Correlation of thoracic width being marginally better than cardiac width r = 0.95 (95% C.I. 0.93–0.97) versus r = 0.93 (95% C.I. 0.90–0.95) respectively. Agreement between the 2 readers in classification of cardiomegaly (CTR≥0.5) by kappa statistic was substantial **κ** = 0.78 (95% C.I 0.61–0.95) (Fig 2).

### Reproducibility of CTR

CXR were repeated in 51 participants after a median of 48 days (IQR 40–72), CTR measurements performed by reader 1 at the 2 time points were highly correlated r = 0.77 (95% C.I 0.68–0.88) and agreement of cardiomegaly (CTR≥0.5) was excellent **κ** = 0.92 (95% C.I. 0.77–1) (Fig 2). Of the 51 with repeat CXR, 12 participants had repeat CXR performed on a different x-ray machine, and CTR remained highly correlated r = 0.89 (95% C.I 0.66–0.97) (Fig 2).

### Factors associated with CTR≥0.50

Table 1 shows baseline characteristics of patients categorised by cardiomegaly using a CTR cut-off of 0.50. There was no difference in the prevalence categorising by gender or ART status. The median age was higher in patients with cardiomegaly compared to persons without (36.5 years; IQR (32–41) vs. 34 years; IQR (30–39); p = 0.04). Patients with cardiomegaly had a higher median body mass index (BMI) (31.2 kg/m^2^; IQR 27.3–38.2 versus 26.9 kg/m^2^; IQR 23.2–32.4; p<0.0001) and had higher systolic blood pressures (130mmHg; IQR 121–141 versus 125mmHg; IQR 117–135; p = 0.01). Comparing existing co-morbidities risk factors, there was a higher prevalence of hypertension (16.7% vs. 5.2%, p = 0.001) and diabetes mellitus (5.6% vs. 1.3%, p = 0.029) in participants with cardiomegaly (Table 1). Multivariate logistic regression was subsequently carried out and a model including BMI > 25, history of stroke, Diabetes mellitus and observed or reported hypertension as explanatory variables were found to best predict cardiomegaly (Table 2). Age was found to be associated with hypertension and inclusion did not improve the model.

**Table 1:** Association of variable and risk factors with cardiomegaly—Table compares participants with and without cardiomegaly for a number of variables and risk factors. Med = median, IQR = Interquartile Range, VL = viral load.

Variable	CTR ≥0.50: n = 57	CTR <0.50: n = 393	p-value
Age (years)—Med(IQR): n = 450	36 (32–41)	34 (30–39)	0.04
BMI (kg/m^2^)—Med(IQR): n = 441	31.3 (27.4–37.4)	26.9 (23.2–32.4)	<0.0001
Systolic BP (mmHg)—Med(IQR): n = 437	130 (121–141)	125 (117–135)	0.01
Pulse (bpm)—Med(IQR): n = 441	69 (65–77)	72 (66–81)	0.12
Diastolic BP (mmHg)—Med(IQR): n = 437	81 (75–91)	79 (72–87)	0.08
CD4 count (/mm^3^)—Med (IQR): n = 449	639 (461–757)	554 (442–720)	0.22
VL if not on ART (Copies/mL)—Med (IQR): n = 135	9983 (2703–18011)	7734 (1955–25401)	0.24
Days since HIV diagnosis—Med (IQR): n = 437	1937 (1020–3291)	1934 (973–2869)	0.56
Reported hypertension (%): n = 439	16.7%	5.2%	0.001
Diabetes mellitus (%): n = 438	5.6%	1.3%	0.029
History of stroke (%): n = 439	1.9%	0.26%	0.104
On ART (%): n = 450	71.9%	69.7%	0.734
Current or Ex-Smoker (%): n = 446	10.9%	22.0%	0.057
Current Alcohol consumption (%): n = 446	18.2%	27.6%	0.137
Female (%): n = 450	89.5%	86.0%	0.471

**Table 2:** Results of multivariable logistic regression.

Variable	p-value	Odds Ratio (95% C.I)
BMI (>25kg/m^2^)	<0.001	5.8 (2.2–15.3)
Hypertension (observed or reported)	0.004	2.4 (1.3–4.5)
History of stroke	0.031	26.7 (1.4–529.7)
Diabetes mellitus	0.047	5.6 (1.0–30.3)

## Discussion

We have shown that correlation of CTR measurement between readers was extremely high and that there was a substantial agreement in the classification of cardiomegaly (CTR≥0.50). Furthermore we have demonstrated that over a period of 6–10 weeks, CTR is a stable measurement with high correlation and excellent agreement in classification of cardiomegaly when measured by the same reader. CTR is therefore a robust and reproducible measurement and it would be appropriate to further evaluate its utility as a screening tool for cardiovascular abnormalities in this population.

Our finding that inter-observer agreement for CTR is high is in keeping with previous findings[18, 19]. However to our knowledge, this is the first study to show that CTR is stable over time, suggesting that cardiac motion and subtle alterations in positioning and depth of inspiration do not have a significant impact on CTR. In addition to this, we have shown that CTR is reproducible using different X-Ray machines.

We found 12.7% of our HIV-1 infected outpatient population of predominantly women aged 30–40 had evidence of cardiomegaly. Those with cardiomegaly were more likely to have elevated BMI and systolic blood pressure, in keeping with previous studies[9]. We did not find any differences in prevalence of cardiomegaly between those on ART and those that were ART-naïve. However, our study was not specifically designed to address this and duration and type of ART may have an impact on cardiovascular disorders.

Performance characteristics of CTR as a screening tool to identify HIV-associated heart disease have yet to be defined. However, a recent systematic review evaluating CTR to predict LV dilatation on echocardiogram looked at 6 studies (none of which focused on HIV or an African setting) with a total of 466 patients, CTR ≥0.5 had sensitivity 83.3%, specificity 42.5%, positive predictive value 43.5% and negative predictive value 82.7% for LV dilatation on echocardiogram[20]. The high sensitivity and negative predictive value are important characteristics of a screening test. Although those with LV dilatation and normal CTR might be missed by the use of CXR screening, elevated CTR has been shown to be a predictor of mortality in those with dilated cardiomyopathy suggesting those at highest risk of mortality would likely be identified[21]. Low specificity and positive predictive value increase number needed to screen, which may impact on resources and patient anxiety. However in the context of HIV associated cardiac disease a number or other important conditions may lead to increased CTR in particular pericardial effusion and pulmonary hypertension as well as conditions not specifically associated with HIV but common in SSA such as rheumatic heart disease and hypertensive heart disease. This would result in an improvement in positive predictive value for significant cardiac disorders.

It is also possible that there may be certain groups where sensitivity of CTR may be reduced, for example those with chronic obstructive pulmonary disease (COPD), usually secondary to smoking, often have increased thoracic diameter and therefore a reduced CTR. In our study we did not find a significant difference in prevalence of cardiomegaly between smokers and non-smokers, although our participants were relatively young and therefore COPD would be expected to be uncommon. Ultimately, CTR alone may not have the desired sensitivity and specificity required to screen for cardiovascular disorders in all populations. It may be that incorporation of additional measures such as electrocardiogram, symptom screen or evaluation of exercise tolerance into a screening algorithm are required to optimize performance. Further studies will be needed to investigate this.

Our study has several limitations; we only evaluated a single approach to measurement of CTR. Measurement of CTR is not standardised with several approaches described for both measurement of cardiac and thoracic width and furthermore other measurements of cardiac size on CXR are possible. In the original method described by Danzer, maximal thoracic width was used but since then others have used thoracic width at the level of the right hemi-diaphragm, left hemi-diaphragm or right costophrenic angle[22, 23], thoracic diameter can vary up-to 3.9% depending on methodology used. We chose to measure maximal thoracic diameter and therefore may have slightly underestimated CTR[18]. Although CTR is the most widely used measurement of cardiac size that can be made on CXR, it is not the only useful measurement. Transverse diameter of the heart shadow on CXR alone has been shown to be more highly correlated (r = 0.75) with LVEDV, determined by cardiac MRI, than CTR (r = 0.46)[13]. A more complex calculation of heart volume can also be made using a lateral in addition to a PA view, although this is a slightly more sensitive technique to identify ventricular hypertrophy at autopsy compared with CTR (65% vs 57%)[24].

We did not specifically explore factors that might affect inter-observer variability or reproducibility of the CTR. It is possible patient factors such as presence of epicardial fat pad or significant breast shadow may obscure the cardiac border affecting this. However technical factors may be more important. Our study was conducted in a trial setting and all CXR were performed to a strict standardized operating procedure (SOP) with radiographs of poor technical quality repeated. In a clinical setting there may be greater technical variation between radiographs, which might be expected to affect stability of CTR, as rotation and changes in inspiratory effort might affect reproducibility. In addition, CXR in our study were digital, which allows the reader to optimize contrast of image on a screen prior to taking measurements. In conventional radiography this cannot be done and poorly penetrated films may result in obscuring of the cardiac shadow, which may contribute to uncertainly over the maximal cardiac diameter and result in increased inter-observer variability.

We showed that CTR was reproducible on a repeated image and hence also showed it was stable over time. However, the time period between CXR (median 48 days) was determined by the protocol of the vaccine trial this study was embedded within and so we were unable to evaluate whether CTR would be stable over longer periods of time.

## Conclusion

Screening algorithms to identify HIV-1-infected persons at high risk of cardiovascular disease or needing referral for specialist investigation that can be implemented in resource limited settings care need to be developed. Revisiting how CTR might be useful in this context as a non-invasive marker for identifying structural cardiac abnormalities in HIV-1-infected populations seems warranted. We have shown CTR is a robust and reproducible measure that can be taken forward into future studies.
